# Prognostic significance of platelet–lymphocyte ratio in patients receiving first-line tyrosine kinase inhibitors for metastatic renal cell cancer

**DOI:** 10.1186/s40064-016-3592-4

**Published:** 2016-10-28

**Authors:** Tae Ju Park, Yang Hyun Cho, Ho Seok Chung, Eu Chang Hwang, Sung-Hoon Jung, Jun Eul Hwang, Woo Kyun Bae, Jin Woong Kim, Suk Hee Heo, Young Hoe Hur, Seung Il Jung, Dong Deuk Kwon

**Affiliations:** 1 Department of Urology, Chonnam National University Medical School, 42 Jebongro, Donggu, Gwangju, 501-757 Republic of Korea; 2 Department of Hemato-Oncology, Chonnam National University Medical School, Gwangju, Republic of Korea; 3 Department of Radiology, Chonnam National University Medical School, Gwangju, Republic of Korea; 4 Department of Hepato-Pancreato-Biliary Surgery, Chonnam National University Medical School, Gwangju, Republic of Korea

**Keywords:** Platelet–lymphocyte ratio, Neutrophil–lymphocyte ratio, Neoplasm metastasis, Carcinoma, renal cell

## Abstract

**Background:**

The platelet–lymphocyte ratio (PLR) and neutrophil–lymphocyte ratio (NLR) have been reported as prognostic factors in various cancers, but their roles in metastatic renal cell cancer (mRCC) remain unclear. We investigated the significance of PLR and NLR, along with that of established prognostic factors, in mRCC patients receiving first-line tyrosine kinase inhibitors (TKI).

**Methods:**

Data obtained from 63 mRCC patients who received first-line TKI between 2007 and 2013 were evaluated retrospectively. The association of PLR, NLR, and established prognostic factors with progression-free survival (PFS) and overall survival (OS) was analyzed using the Kaplan–Meier method. The influence of independent prognostic factors on survival was determined using multivariable Cox regression analysis.

**Results:**

High NLR (>3.6) and PLR (>150) were related to shorter PFS (p = 0.001) and OS (p = 0.001). The presence of brain metastases [hazard ratio (HR) 4.94, 95% CI 1.75–13.9; p = 0.002] and high PLR (>150, HR 13.1, 95% CI 5.14–33.2; p = 0.001) were independently associated with PFS, and Eastern Cooperative Oncology Group Performance status ≥2 (HR 3.60, 95% CI 1.39–9.31; p = 0.008), lymph node metastasis (HR 2.76, 95% CI 1.11–6.86; p = 0.029), brain metastasis (HR 9.39, 95% CI 2.74–32.1; p = 0.001), and high PLR (>150, HR 16.1, 95% CI 4.41–58.4; p = 0.001) with OS.

**Conclusions:**

High PLR was associated with shorter survival of mRCC patients receiving first-line TKI. The PLR may be an effective independent prognostic factor in this setting.

## Background

Renal cell carcinoma (RCC) is the most common type of kidney cancer in adults. It accounts for approximately 3% of adult malignancies and 90–95% of neoplasms arising from the kidney. Patients with RCC progress to a metastatic stage in approximately 33% of cases (Gunduz et al. 2015). The management of patients with metastatic RCC (mRCC) is complicated by the lack of proven effectiveness of accessible therapies. Recently, tyrosine kinase inhibitors (TKI) such as sunitinib, sorafenib, or pazopanib have become a common choice for first-line targeted treatment of mRCC (Noronha et al. 2016). Common metastatic sites include the lung, liver, bones, brain, and adrenal gland, while many case reports describe the capacity of RCC to metastasize almost anywhere in the body. More than one organ system is often affected by the metastatic process. Metastases may be found at the time of diagnosis or at any point after nephrectomy (Thyavihally et al. 2005). About 20–50% of patients will eventually develop a metastatic disease after nephrectomy. A shorter time interval between nephrectomy and the development of metastases is associated with a poor prognosis. Also, the presence of biological signs of inflammation, shorter time interval between diagnosing a renal tumor and developing metastases (<1 year), elevated neutrophil counts, liver metastases, bone metastases, patient performance status (PS), the number of metastatic sites, alkaline phosphatase and hemoglobin levels are predictive factors of survival outcome (Heng et al. 2009). The crucial role of microenvironment inflammation in the development and progression of malignancies by influencing the proliferation and survival of tumor cells, promoting angiogenesis and metastasis, and reducing responses to anti-cancer agents has been recognized (Gunduz et al. 2015; de Vivar Chevez et al. 2014). Recently, several biomarkers and hematological indices representative of systemic inflammatory responses, including the neutrophil–lymphocyte ratio (NLR), the platelet–lymphocyte ratio (PLR), and the combination of the C-reactive protein (CRP) and albumin known as the Glasgow prognostic score (GPS) were introduced as prognostic factors in various cancers. For example, the NLR and PLR have been suggested as prognostic indicators for lung, colorectal, breast, gastric and pancreatic cancer, as well as urinary cancers such as RCC, and urothelial carcinoma (Feng et al. 2014; Raungkaewmanee et al. 2012; Hu et al. 2015; Song et al. 2016; Nakano et al. 2014). Templeton et al. (2014a, b) investigated the prognostic role of NLR and PLR in solid tumors. In their meta-analyses, both high NLR and PLR were associated with adverse overall survival (OS). Moreover, the effect of high PLR on OS was greater for metastatic disease than for early stage disease (Templeton et al. 2014b). However, Keskin et al. (2014) showed that only preoperative high NLR was associated with mortality in renal cell carcinoma. Thus, the aim of this study was to investigate the relationship of NLR, PLR, and previously known prognostic factors with progression-free (PFS) and OS in patients with mRCC.

## Methods

### Patients

The data of 63 patients who had received first-line TKI for mRCC between 2007 and 2013 were reviewed retrospectively. Patients with histologically confirmed mRCC (all clear cell histology) who had received first-line sunitinib or pazopanib according to the standardly approved schedule and dosage were eligible for this study. Sunitinib was administered at a dose of 50 mg per day for 4 weeks, followed by a 2-week rest. Pazopanib was administered at a dose of 800 mg per day, continuously during 4 weeks. Both TKIs were prescribed strictly as monotherapy, in either standard everyday clinical practice or as a part of a clinical trial. Dose reduction was performed in a case of toxicity, according to standard recommendations for the two agents. Before treatment, the patient’s age, sex, clinical symptoms, medical history, and performance status were evaluated by physicians. Blood samples were collected for serum chemistry and hematological testing. Chest, abdominal and pelvic computed tomography, and positron emission tomography–computed tomography were also performed. Responses were evaluated every 8–12 weeks after the treatment initiation by chest and abdominopelvic computed tomography or by the same tests that were used to stage initial tumors. TKI therapy was given until objective disease progression per Response Criteria in Solid Tumors v. 1.1 criteria (Eisenhauer et al. 2009). PFS was defined as the period of time between the initial administration of a treatment and the first detection of tumor progression based on radiological criteria or death. OS was calculated from the start of treatment to the date of any cause of death.

### Assessment of Eastern Cooperative Oncology Group Performance status (ECOG-PS), NLR and PLR

ECOG-PS was assessed at the time of diagnosis. Data on pretreatment blood cell counts were extracted in a retrospective fashion from the medical records. White blood cell and differential counts were assessed just prior to administration of TKIs. The NLR was defined as the absolute neutrophil count divided by the absolute lymphocyte count, and was categorized into two groups (≤3.6 and >3.6). Similarly, PLR was defined as the absolute platelet count divided by the absolute lymphocyte count, and was also categorized into two groups (≤150 and >150). The cut-off values were evaluated using the receiver operating characteristic (ROC) curve.

### Statistical analysis

Statistical analysis was conducted using IBM SPSS software package version 21.0 (Statistical Package for Social Sciences, IBM, Armonk, NY) and MedCalc software version 15 (MedCalc Software bvba, Ostend, Belgium). The PFS and OS were calculated using the Kaplan–Meier method. Multivariable analyses using the Cox regression proportional hazard model were performed to evaluate the prognostic significance of all parameters on PFS and OS. ROC curves were plotted to verify the accuracy of the obtained significance of NLR and PLR on overall survival prediction. A *p* value < 0.05 was considered as statistically significant.

## Results

### Clinical characteristics

The clinical characteristics of 63 patients who received first-line TKI for mRCC are summarized in Table 1.

**Table 1:** Patient demographics

Patient no., n	63
Age, median (IQR)	63.1 (56.0–70.5)
Median follow up, months (IQR)	17.5 (9.2–28.4)
Sex, n (%)	
Male	52 (82.5)
Female	11 (17.5)
ECOG PS, n (%)	
0–1	36 (57.1)
≥2	27 (42.9)
Disease free interval, n (%)	
<1 year	40 (63.5)
≥1 year	23 (36.5)
Lung metastasis, n (%)	
Yes	39 (61.9)
No	24 (38.1)
Liver metastasis, n (%)	
Yes	6 (9.5)
No	57 (90.5)
Lymph node metastasis, n (%)	
Yes	25 (39.7)
No	38 (60.3)
Bone metastasis, n (%)	
Yes	19 (30.2)
No	44 (69.8)
Brain metastasis, n (%)	
Yes	5 (7.9)
No	58 (92.1)
Metastasis at other site, n (%)	
Yes	13 (20.6)
No	50 (79.4)
Number of metastatic sites, n (%)	
1	37 (54.4)
≥2	31 (45.6)
Previous nephrectomy, n (%)	
Yes	35 (55.6)
No	28 (44.4)
TKI regimen, n (%)	
Sunitinib	40 (63.5)
Pazopanib	23 (36.5)
Response to TKIs, n (%)	
CR	3 (4.8)
PR	12 (19.0)
SD	35 (55.6)
PD	13 (20.6)
Neutrophil–lymphocyte ratio, n (%)	
≤3.6	34 (54.0)
>3.6	29 (46.0)
Platelet–lymphocyte ratio, n (%)	
≤150	37 (58.7)
>150	26 (41.3)
Hemoglobin, n (%)	
<12 (g/dL)	25 (39.7)
≥12 (g/dL)	38 (60.3)
Corrected calcium, n (%)	
<10 (mg/dL)	57 (90.5)
≥10 (mg/dL)	6 (9.5)
MSKCC risk	
Favorable	19 (30.2)
Intermediate	41 (65.1)
Poor	3 (4.8)
Progression	48 (76.2)
Death	29 (46.0)

*IQR* Interquartile range, *ECOG PS* Eastern cooperative oncology group performance status, *TKI* Tyrosine kinase inhibitor, *CR* complete response, *PR* partial response, *SD* stable disease, *PD* progressive disease, *MSKCC* Memorial Sloan Kettering Cancer Center

The group consisted of 52 men and 11 women, with a median age of 63 [interquartile range (IQR), 56.0–70.5] years. The median follow up period was 17.5 months (IQR 9.2–28.4 months). Thirty-five (55.6%) patients had undergone previous nephrectomy, 36 (57.1%) patients had ECOG PS < 2, and 40 (64%) patients had developed metastases within 1 year. Nineteen patients had bone metastases, 39 had lung metastases, 6 had liver metastases, 25 had lymph node metastases, 5 had brain metastases and 31 patients presented with multiple metastatic sites. Hemoglobin level of <12 was found in 25 (39.7%) patients, corrected calcium level of <10 in 57 (90.5%) patients, 48 (76.2%) patients had tumor progression, and 29 (46.0%) patients died. In addition, most patients had favorable (n = 19, 30.2%) or intermediate (n = 41, 65.1%) risk as determined by Memorial Sloan Kettering Cancer Center criteria (Motzer et al. 1999).

Values of NLR > 3.6 and PLR > 150 were obtained in 29 (46.0%) and 26 (41.3%) patients, respectively. Sunitinib was administered to 40 patients and pazopanib to 23 patients. Four types of response to TKIs were defined: complete response (CR), partial response (PR), stable disease (SD) and progressive disease (PD), which were detected in 3 (4.8%), 12 (19.0%), 35 (55.6%) and 13 (20.9%) cases, respectively. Finally, 31 patients received second line treatments using everolimus after TKI failure.

### ROC curves for NLR and PLR

**Fig. 1:**
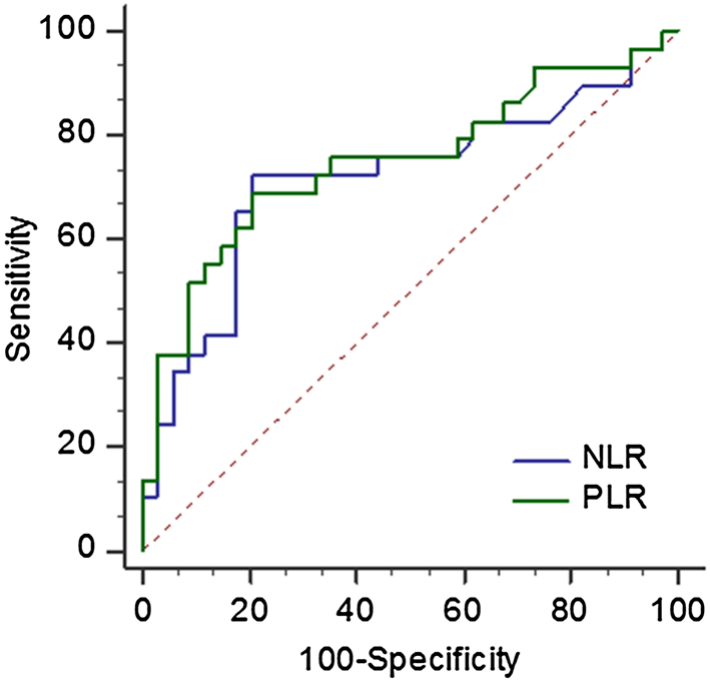
Receiver operator characteristic curves for the neutrophil-lymphocyte ratio (NLR) and the platelet–lymphocyte ratio (PLR). For NLR: area under the curve = 0.72, p = 0.002, sensitivity = 72.4%, specificity = 76.4%, accuracy = 74.6%. For PLR: area under the curve = 0.75, p = 0.001, sensitivity = 68.9%, specificity = 79.4%, accuracy = 73.0%

The areas under the ROC curves for NLR and PLR were 0.72 (p = 0.002) and 0.75 (p = 0.001), respectively (Fig. 1). However, the difference between the NLR and PLR values was not significant (p = 0.620).

### The effect of clinical variables and prognostic factors on PFS and OS

The median PFS and OS were 10.3 months (95% CI 8.7–11.9) and 31.9 months (95% CI 14.2–49.5), respectively. The results of the Kaplan–Meier analysis of the effect of clinical parameters on PFS and OS are shown in Table 2. Shorter PFS was associated with an ECOG score ≥2, the presence of brain metastases, ≥2 metastatic sites, NLR > 3.6 and PLR > 150 (Table 2, all p < 0.05 values). Multivariable analysis showed that brain metastases (HR 4.94, 95% CI 1.75–13.9; p = 0.001) and PLR > 150 (HR 13.1, 95% CI 5.14–33.2; p = 0.001) were independent prognostic factors of PFS (Table 3).

**Table 2:** Results of the Kaplan–Meier analysis of progression free and overall survival

	PFS, months (95% CI)	p value	OS, months (95% CI)	p value
Age				
<64 years	10.3 (8.4–12.2)	0.714	31.9 (11.7–52.1)	0.751
≥64 years	10.4 (6.7–14.1)		27.1 (5.3–48.8)	
Sex				
Male	10.6 (7.5–13.6)	0.245	31.9 (12.2–51.6)	0.936
Female	10.1 (5.8–14.2)		25.8 (8.2–43.4)	
ECOG PS				
0–1	15.1 (9.2–20.9)	0.001	63.5 (30.8–96.2)	0.001
≥2	8.2 (3.4–12.9)		15.1 (10.7–19.5)	
Disease free interval				
<1 year	9.2 (5.1–13.3)	0.063	22.0 (0–50.5)	0.131
≥1 year	13.2 (9.2–17.2)		41.1 (16.1–65.9)	
Lung metastasis				
Yes	6.9 (2.0–11.7)	0.139	27.1 (18.4–35.7)	0.722
No	13.2 (6.7–19.7)		37.2 (12.7–61.7)	
Liver metastasis				
Yes	4.3 (0.0–11.1)	0.680	14.4 (0–49.1)	0.388
No	10.4 (8.9–12.0)		31.9 (15.8–48.0)	
Lymph node metastasis				
Yes	10.3 (7.6–13.0)	0.180	14.4 (9.1–19.7)	0.037
No	10.4 (7.9–12.9)		40.3 (28.4–52.1)	
Bone metastasis				
Yes	10.1 (6.6–13.4)	0.309	18.2 (13.3–23.1)	0.021
No	10.6 (8.8–12.3)		40.3 (20.2–60.3)	
Brain metastasis				
Yes	3.2 (2.1–4.3)	0.001	5.9 (0.1–11.7)	0.012
No	10.6 (9.0–12.1)		37.2 (17.1–57.3)	
Metastasis at other site				
Yes	9.1 (5.8–12.5)	0.620	27.1 (10.7–43.4)	0.384
No	10.4 (8.4–12.4)		63.5 (6.2–120.8)	
Number of metastatic site				
1	13.2 (8.7–17.7)	0.001	40.3 (24.7–55.8)	0.007
≥2	5.0 (0.4–9.6)		13.7 (9.3–18.1)	
Previous nephrectomy				
Yes	10.5 (6.0–15.1)	0.522	37.2 (19.1–55.3)	0.113
No	10.3 (7.1–13.5)		18.2 (6.6–29.7)	
TKI regimen				
Sunitinib	10.1 (7.6–12.5)	0.664	37.2 (15.9–58.5)	0.786
Pazopanib	10.6 (10.1–11.1)		25.8 (10.2–41.3)	
Neutrophil–lymphocyte ratio				
≤3.6	16.3 (10.4–22.2)	0.001	63.5 (37.4–89.6)	0.001
>3.6	5.0 (0.7–9.3)		17.0 (12.4–21.6)	
Platelet–lymphocyte ratio				
≤150	18.1 (6.2–29.9)	0.001	50.5 (28.5–72.4)	0.001
>150	4.4 (3.5–5.3)		13.7 (7.8–19.6)	
Hemoglobin				
<12 (g/dL)	7.2 (0.3–13.9)	0.237	25.8 (8.3–43.2)	0.252
≥12 (g/dL)	11.7 (8.6–14.8)		37.2 (17.5–56.8)	
Corrected calcium				
<10 (mg/dL)	10.3 (8.7–11.9)	0.652	31.9 (14.4–49.4)	0.206
≥10 (mg/dL)	Not reached		Not reached

**Table 3:** Results of the multivariable analysis of variables affecting progression free and overall survival

	Progression free survival		Overall survival	
	HR	p value	HR	p value
ECOG PS (≥2)			3.60 (1.39–9.31)	0.008
Lymph node metastasis			2.76 (1.11–6.86)	0.029
Brain metastasis	4.94 (1.75–13.9)	0.002	9.39 (2.74–32.1)	0.001
Platelet–lymphocyte ratio (>150)	13.1 (5.14–33.2)	0.001	16.1 (4.4–58.4)	0.001

*ECOG PS* Eastern cooperative oncology group performance status, *HR* Hazard ratio

Meanwhile, ECOG PS ≥ 2, lymph node metastasis, bone metastasis, brain metastasis, number of metastatic sites ≥2, NLR > 3.6, and PLR > 150 were related to shorter OS (Table 2, all p < 0.05). Also, ECOG PS ≥ 2 (HR 3.60, 95% CI 1.39–9.31; p = 0.008), lymph node metastasis (HR 2.76, 95% CI 1.11–6.86; p = 0.029), brain metastasis (HR 9.39, 95% CI 2.74–32.1; p = 0.001), and PLR > 150 (HR 16.1, 95% CI 4.4–58.4; p = 0.001) were independent prognostic factors of OS (Table 3).

## Discussion

Guidelines for the treatment of mRCC are rapidly evolving to incorporate the recently approved molecular-targeted therapies. Many mRCC patients have a poor prognosis, and TKIs have extremely changed their prospects. Prognostic factors can help clinicians determine the most appropriate use of TKIs by selecting the patients most likely to benefit from them (Noronha et al. 2016). Although numerous studies have attempted to determine which clinical factors might be predictors of PFS and OS, the results were inconsistent (Heng et al. 2009; Raungkaewmanee et al. 2012). In this study, we analyzed data obtained from 63 mRCC patients receiving first-line TKI with the aim of performing prognostic evaluations and promoting optimal surveillance strategies. Many studies have proved that a poor ECOG PS remains one of the most significant prognostic factors for various malignancies, such as GI cancer, lung cancer, ovarian cancer and mRCC (Zimmermann et al. 2010). Patients with a worse ECOG PS and limited functional capacity tend to tolerate cancer treatments with more difficulty. These patients have less favorable outcomes than patients with a better PS. This has been further strengthened by similar conclusions obtained from this study. However, one of the challenges when using PS as a prognostic measure is that it is subjective and may not be reproducible (Laird et al. 2013), as it is difficult to determine it accurately in the busy clinical setting, relying heavily on patient-reported information. In 1999, the Memorial Sloan-Kettering Cancer Center (MSKCC) reported for the first time that hemoglobin level lower than the normal limit, corrected calcium level higher than the normal limit, Karnofsky performance status lower than 80% and less than 1 year interval from diagnosis to treatment were independent predictors of short survival (Motzer et al. 1999; Cetin et al. 2014). This study defined pretreatment clinical features that were prognostic of survival in previously untreated mRCC patients who subsequently received cytokine therapy, such as IFN-α. Heng et al. (2009) also reported that lower hemoglobin level and higher serum corrected calcium, poor PS, and less than 1 year interval from initial diagnosis to initiation of therapy were independent predictive factors of poor survival. However, our study showed that hemoglobin and calcium levels were not significantly associated with the prognosis outcome of mRCC patients. Vickers et al. (2013) suggested that the number of brain metastases is also a prognostic factor of OS. Our study also found that the presence of brain metastases is associated with poor OS, while no significant correlation with PFS was observed. Kroeger et al. (2015) reported that the presence of lymph node metastases below the diaphragm is associated with poor survival in mRCC patients treated with targeted therapies. Our results are consistent with theirs concerning OS, but not PFS. The prognostic role of previous nephrectomy has recently been reported in patients with mRCC treated with target therapy (Choueiri et al. 2011; de Groot et al. 2016). Our results suggest that previous nephrectomy was not a significant prognostic factor for mRCC patients. All these differences may be due to the small number of analyzed mRCC patients and the characteristics of the study group, thus, further studies are needed.

Since Lu et al. (2006) suggested that tumors might form as a consequence of chronic inflammation, many studies have confirmed that inflammation is crucial for the development and progression of cancer. Therefore, inflammation markers have been regarded as significant for tumor progression and as risk factors for tumor recurrence. It has been recently suggested that systemic inflammation-based scores such as GPS (including albumin and CRP values), NLR, and PLR might be significant predictors of OS and PFS of cancer patients (de Vivar Chevez et al. 2014; Feng et al. 2014; Raungkaewmanee et al. 2012; Hu et al. 2015; Song et al. 2016; Nakano et al. 2014; Templeton et al. 2014a, b; Keskin et al. 2014). Over the last decade, it has become clear that these parameters are consistently associated with poor outcome, regardless of the tumor stage. Several studies suggested that inflammation is the key component of the tumor microenvironment, and that it has a significant role in carcinogenesis and disease progression (Balkwill and Mantovani 2012). Recent studies have tried to shed new light on molecular and cellular pathways linking inflammation and cancer (Porta et al. 2009). Despite the fact that RCC is an immunogenic tumor, there is little evidence that immune cells and inflammatory pathways can enhance the tumor’s growth and immune escape. However, recent studies are beginning to uncover the mechanisms of immune escape in RCC, and the role of inflammatory immune cells and cytokines is this process (Gunduz et al. 2015; de Vivar Chevez et al. 2014). Tumor-associated neutrophils (TANs) have a significant role in the malignant setting, as they may act as potent antitumor effector cells. However, increasing clinical evidence shows that TANs correlate with poor prognosis. As the tumor microenvironment controls neutrophil recruitment, TANs help tumor progression (Uribe-Querol and Rosales 2015). Reduction of lymphocyte infiltration in cancer-adjacent tissues tends to lead to the proliferation and metastasis of tumor cells (Whiteside 2014). Platelets can also lead to a higher level of cancer-related coagulation (Falanga et al. 2013). It is possible that active systemic inflammation induces a poor local immune response to a tumor, leading to lymph node spread and metastasis. Therefore, the ratios of platelets, neutrophils, and lymphocytes might be important prognostic factors in various cancers. Raungkaewmanee et al. (2012) studied the predictive value of PLR and NLR on the outcome of epithelial ovarian cancer patients. They reported that higher pretreatment PLR led to shorter PFS and OS, and that PLR was a better prognostic predictor of survival than NLR. However, Chrom et al. (2016) showed that only NLR ≥ 4 was significantly associated with shorter survival, while PLR ≥ 200 tended to associate with poor survival. Moreover, recent study revealed that high NLR at baseline and high NLR after targeted therapy was associated with shorter PFS and OS in mRCC (Templeton et al. 2016). However, this study investigated the effect of NLR on PFS and OS without other prognostic factors. Although our study included relative small patients, we evaluated the effect of PLR on PFS and OS in mRCC in addition to NLR and other prognostic factors. The data obtained in this study are not entirely in accordance with available literature data. We found that ECOG PS ≥ 2, brain metastases, multiple metastases, high NLR (>3.6), and high PLR (>150) were predictors of shorter PFS and poor OS when performing a Kaplan–Meier analysis. However, only the presence of brain metastases and a high PLR (>150) were found to be significant prognostic factors in terms of PFS and OS when performing the multivariable analysis. Our study has several limitations. First, it was a retrospective uncontrolled study regarding a single center’s experience with a relatively uncommon disease, thus, the number of analyzed patients was quite small. Second, the follow-up period might not have been long enough, so a further randomized clinical study involving more mRCC patients is needed. Third, the NLR and PLR cutoff values have not been previously established and were selected from the ROC curves. Fourth, we did not assess underlying disorders and other variables (e.g., diabetes mellitus, hypertension, body mass index, smoking, diet, nutrition) of the patients. Finally, our findings were expressed in terms of PFS and OS, rather than cancer specific survival. However, an inflammation-based prognostic score is a simple, convenient, and easily determined parameter that clinicians can use for assessing the prognosis of mRCC patients.

## Conclusions

Our results suggest that a high PLR (>150) and brain metastases were associated with shorter PFS and poor OS in patients with mRCC. In addition, higher PS (≥2) and lymph node metastases were associated with shorter OS. As PLR can be determined by readily available and inexpensive tests, it might serve as an ideal biomarker for predicting the survival of patients with mRCC. Patients with high PLR might require a more appropriate treatment than first-line TKI such as immunotherapy using PD-1/PD-L1 inhibitors, but a larger prospective and multi-institutional study is needed in order to confirm these data.

## Abbreviations

PLR: platelet–lymphocyte ratio
NLR: neutrophil–lymphocyte ratio
mRCC: metastatic renal cell cancer
TKI: tyrosine kinase inhibitors
CRP: C-reactive protein
PFS: progression-free survival
OS: overall survival
HR: hazard ratio
PS: performance status
